# Experience-Dependent Coding of Time-Dependent Frequency Trajectories by Off Responses in Secondary Auditory Cortex

**DOI:** 10.1523/JNEUROSCI.2665-19.2020

**Published:** 2020-06-03

**Authors:** Kelly K. Chong, Dakshitha B. Anandakumar, Alex G. Dunlap, Dorottya B. Kacsoh, Robert C. Liu

**Affiliations:** ^1^Wallace H. Coulter Department of Biomedical Engineering, Georgia Institute of Technology and Emory University, Atlanta, Georgia 30332; ^2^Department of Biology, Emory University, Atlanta, Georgia 30322; ^3^Center for Translational Social Neuroscience, Emory University, Atlanta, Georgia 30322

**Keywords:** belt, maternal behavior, offset response, pitch, secondary auditory cortex, USV

## Abstract

Time-dependent frequency trajectories are an inherent feature of many behaviorally relevant sounds, such as species-specific vocalizations. Dynamic frequency trajectories, even in short sounds, often convey meaningful information, which may be used to differentiate sound categories. However, it is not clear what and where neural responses in the auditory cortical pathway are critical for conveying information about behaviorally relevant frequency trajectories, and how these responses change with experience. Here, we uncover tuning to subtle variations in frequency trajectories in auditory cortex of female mice. We found that auditory cortical responses could be modulated by variations in a pure tone trajectory as small as 1/24th of an octave, comparable to what has been reported in primates. In particular, late spiking after the end of a sound stimulus was more often sensitive to the sound's subtle frequency variation compared with spiking during the sound. Such “Off” responses in the adult A2, but not those in core auditory cortex, were plastic in a way that may enhance the representation of a newly acquired, behaviorally relevant sound category. We illustrate this with the maternal mouse paradigm for natural vocalization learning. By using an ethologically inspired paradigm to drive auditory responses in higher-order neurons, our results demonstrate that mouse auditory cortex can track fine frequency changes, which allows A2 Off responses in particular to better respond to pitch trajectories that distinguish behaviorally relevant, natural sound categories.

**SIGNIFICANCE STATEMENT** A whistle's pitch conveys meaning to its listener, as when dogs learn that distinct pitch trajectories whistled by their owner differentiate specific commands. Many species use pitch trajectories in their own vocalizations to distinguish sound categories, such as in human languages, such as Mandarin. How and where auditory neural activity encodes these pitch trajectories as their meaning is learned but not well understood, especially for short-duration sounds. We studied this in mice, where infants use ultrasonic whistles to communicate to adults. We found that late neural firing after a sound ends can be tuned to how the pitch changes in time, and that this response in a secondary auditory cortical field changes with experience to acquire a pitch change's meaning.

## Introduction

How the brain processes natural stimuli and acquires their meaning is a fundamental question in sensory neuroscience. In audition, species-specific vocalizations are a particularly important natural sound category. Vocalizations can convey meaning through their time-dependent frequency trajectory in a way that is conserved across species (Morton, 1977; Klump and Shalter, 1984; Fischer et al., 1995; Watwood et al., 2004; Brudzynski, 2007; Taylor et al., 2009) and also learned through experience (Ehret et al., 1987; Jouventin et al., 1999; Hollén and Manser, 2006; Razak et al., 2008; Gustafsson et al., 2013). How the auditory system encodes these frequency trajectories as a basis for discriminating vocal categories is thus of great interest.

At the auditory cortical level, responses to nonconstant frequency trajectories have been widely studied in core fields using simple sinusoidal frequency modulations (sFMs) or directional sweeps (Suga, 1964; Whitfield and Evans, 1965; Mendelson and Cynader, 1985; Gaese and Ostwald, 1995; Nelken and Versnel, 2000; Malone et al., 2014). Long-duration stimuli have helped disambiguate the tuning of sustained (Liang et al., 2002) or entrained (Gaese and Ostwald, 1995) and offset (Sollini et al., 2018) components of the response to these modulations. However, many natural vocalizations are short, with complex trajectories over just a single modulation cycle (May et al., 1989; Xu, 1997; Scattoni et al., 2008). What aspects of core or noncore neuronal firing are sensitive to the acoustic parameters of these brief trajectories has not been well explored, leaving open how sound experience affects tuning to these modulations.

We explored in mice the auditory cortical sensitivity to short frequency trajectories, which dominate their species-specific vocalizations (Liu et al., 2003), parameterized by linear frequency modulations (lFM) and sFM. We exploited a mouse maternal model to examine experience-dependent coding of frequency trajectories in natural vocalizations in both the core and a noncore field. Mice come to recognize and prefer approaching pup ultrasonic vocalizations (USVs) after maternal experience (Ehret et al., 1987; Ehret, 2005; Lin et al., 2013). Acquiring the behavioral relevance of pup USVs leads to both inhibitory and excitatory plasticity in the response to USVs within core auditory cortex (Liu et al., 2006; Galindo-Leon et al., 2009; Cohen et al., 2011; Marlin et al., 2015; Shepard et al., 2015; Krishnan et al., 2017). In particular, a specific subset of core neurons comes to discriminate the pup USV category from other vocal categories after experience, even when USVs are matched in their onset frequencies and durations (Shepard et al., 2015). This raises the critical question of whether neurons might be sensitive to systematic differences in how frequency trajectories of different call types unfurl in time, thereby enabling their discrimination (Neilans et al., 2014).

We conducted head-fixed, awake single unit (SU) electrophysiology in both maternal (M) and nonmaternal (Nm) females, and probed plasticity in frequency trajectory sensitivity across natural sound experience and auditory region. We found sensitivity to FM amplitudes (A_fm_) < 1/24th octave, a finer degree of FM sensitivity than previously expected for the mouse. We discovered an enhanced prevalence of responses after the end (Off responses) of natural USVs after maternal experience, specifically in secondary auditory cortex field A2. In maternal A2 SUs, a bias emerged in responses that favored vocalizations with pup-like sFM parameters. This bias coincided with a general shift in tuning to frequency trajectory parameters in the maternal A2 toward values that were more characteristic of the pup USV category. Together, this work suggests that a sensitivity to short frequency trajectories in the Off portion of A2 responses may play a key role in learning the acoustic features of natural vocal categories.

## Materials and Methods

### General methods

Female WT CBA/CaJ mice (RRID:IMSR_JAX:000654) between 12 and 18 weeks of age were used in this study. Animals were socially housed in single-sex cages until breeding age on a 14 h light/10 h dark reverse light cycle with *ad libitum* access to food and water; mice were moved to individual housing during experiments. All animal procedures used in this study were approved by the Emory University Institutional Animal Care and Use Committee.

At 12–18 weeks of age, mice were moved to individual housing, and surgery consisting of headpost attachment followed by small hole craniotomy was conducted (Shepard et al., 2015). Briefly, animals were anesthetized with isoflurane (2%–5%, delivered with oxygen), and buprenorphine (0.1 mg/kg) was administered as an analgesic. Animals underwent aseptic surgery to stereotaxically define a recording grid over the left auditory cortex, as the left auditory cortex is putatively associated with mouse vocalization processing, particularly in the maternal paradigm (Geissler and Ehret, 2004; Marlin et al., 2015). The skull was exposed, and the left temporal muscle was deflected to permit access to the bone overlying auditory cortex. Using sterile tattoo ink applied to a stiff wire mounted on a stereotaxic manipulator, we marked a grid of ∼100-μm-diameter dots on the skull in three rows (1.5, 2.0, and 2.5 mm below bregma) and five columns (spanning 50%–90% of the distance between bregma and λ, in 10% steps). Dental cement was then used to secure an inverted flat-head screw on the midline equidistant from bregma and λ. The animal recovered in the home cage placed on a heating pad and was administered saline subcutaneously for fluid replacement.

The day before a recording, the animal was reanesthetized with isoflurane, holes (∼150 µm in diameter) were hand drilled on one or more grid points, and a ground hole was drilled over the left frontal cortex. The animal was also acclimated to a foam-lined cylindrical (∼3 cm diameter) restraint device, which secured the body while leaving the head exposed. The implanted headpost was then secured to a post mounted on a vibration-isolation table, with the restraint device suspended from rubber bands to keep the body in a comfortable position while reducing torque on the headpost. Recordings typically lasted 2-4 h, and excessive movement or signs of stress signaled the end of an experiment.

Electrophysiological activity in the auditory cortex was recorded on an RX5 data acquisition system (Tucker Davis Technologies, sample rate 24 414.0625/s) in Brainware (Tucker Davis Technologies) with single 6 MΩ tungsten electrodes (FHC), filtered between 300 Hz and either 3 or 6 kHz for SUs. Local field potentials were simultaneously collected from the same electrodes (filtered from 2–300 or 1000 Hz). Using a hydraulic Microdrive (FHC), the electrode was driven orthogonally into auditory cortex to an initial depth of ∼200 µm, and then advanced in 5 µm steps until an SU was detected. SU isolation was based on the absence of spikes during the absolute refractory period (1 ms), and on online cluster analysis of various spike features (e.g., first vs second peak amplitudes, peak-peak times). In some cases, multiple SUs were recorded at one location and could be extracted by clustering based on spike features. Sounds were delivered using Tucker Davis Technologies System 3 hardware, including an RX6 processor (sample rate 223214/s), PA5 programmable attenuator, and SA1 stereo amplifier connected to an Infinity EMIT speaker.

Our recording sites were classified as primary auditory, anterior auditory and ultrasound fields (core) and secondary auditory and dorsoposterior fields (Noncore) according to previously published criteria (Lin et al., 2013; Shepard et al., 2015), following nomenclature used by Stiebler et al. (1997). Briefly, pure tone frequency tuning curves (see below) were derived online for recording sites along a perpendicular penetration into the cortex using the local field potential, which was less variable within a penetration than SU tuning curves (Lin et al., 2013). Core sites were classified based on a combination of factors, including a peristimulus time histogram (PSTH) peak <15 ms from sound onset; and a best frequency (BF) that either fit along the expected tonotopic gradient for A1 and AAF or was likely in ultrasound field (>40 kHz). Penetrations ventral to core sites that were auditory responsive but did not meet these criteria (e.g., longer latency and/or BF that did not fit the tonotopic gradient) were deemed likely A2 sites. In using a stereotaxically specified grid of penetrations (see above), we found good consistency across animals, so that A2 sites were typically situated in the rostral 2 or 3 penetrations in the most ventral row.

### Experimental design and statistical analyses

**Figure 1.**
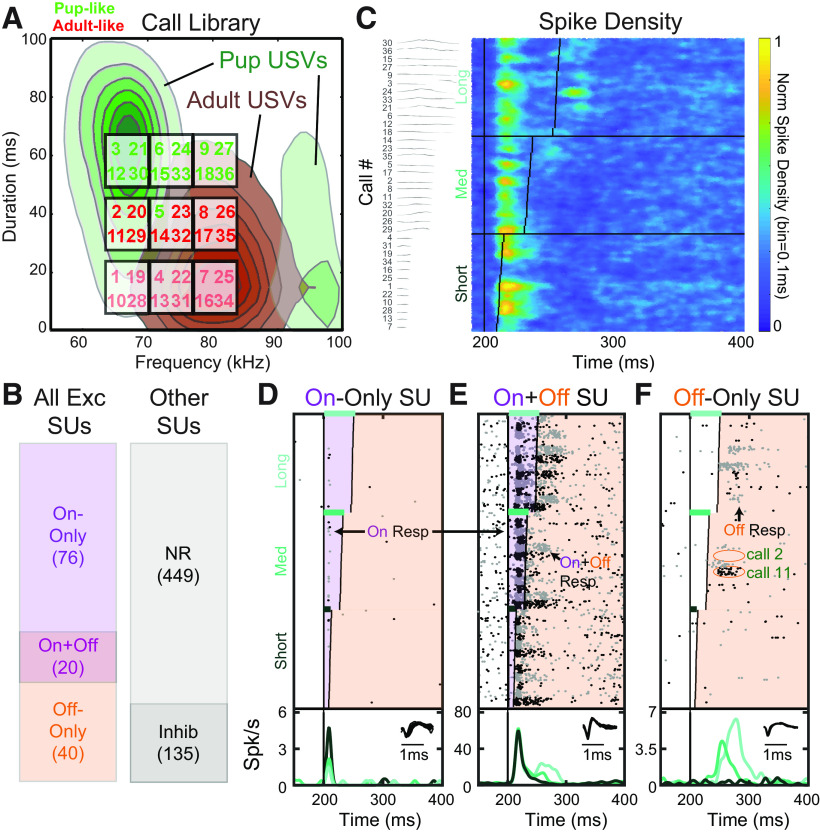
USVs elicit On and Off responses. ***A***, Onset frequency and duration of the ground-truth pup (1–18) and adult (19–36) USVs in our playback library. The underlying distributions for pup (dark green) and adult (dark brown) USVs are shown as contours of increasing likelihood for calls of each ground-truth category to have specific duration and frequency values. Because our calls were chosen to systematically vary in duration and frequency, some actual pup calls are acoustically more similar to adult calls, and vice versa. The bottom row of (adult-like) USVs is shown muted because these are withheld in analyses that required evoked responses to be definitively classified as On or Off, since those calls are too short to do so accurately. ***B***, Overall classification of recorded SUs by their USV-evoked response characteristics. Left, Breakdown of Exc SUs. Right, Breakdown by NR and Inhib, which were not included in subsequent analyses looking at excitatory tuning to USV features. ***C***, Spike density plot depicting overall population responses to USVs across all Exc SUs (*n* = 136, *n* = 55 animals), sorted by increasing USV length. Vertical red lines indicate the duration of each USV, starting with playback at 200 ms. Left column, The USV spectrograms. ***D***, Representative SU with only On responses to several USVs. Rasters alternate between black and gray to delineate the trials of adjacent calls. Purple shaded area represents the On response window. Bottom, The PSTH, which pools responses across all calls within the same duration group: short, medium, and long (rows in Fig. 4*A*). Inset, The SU's spike waveform. ***E***, Similar to ***D***, but for an SU with both On responses and On+Off responses to different USVs. Orange shaded area represents the Off response window. ***F***, Similar to ***C***, but for a SU with only Off responses to several USVs.

**Figure 2.**
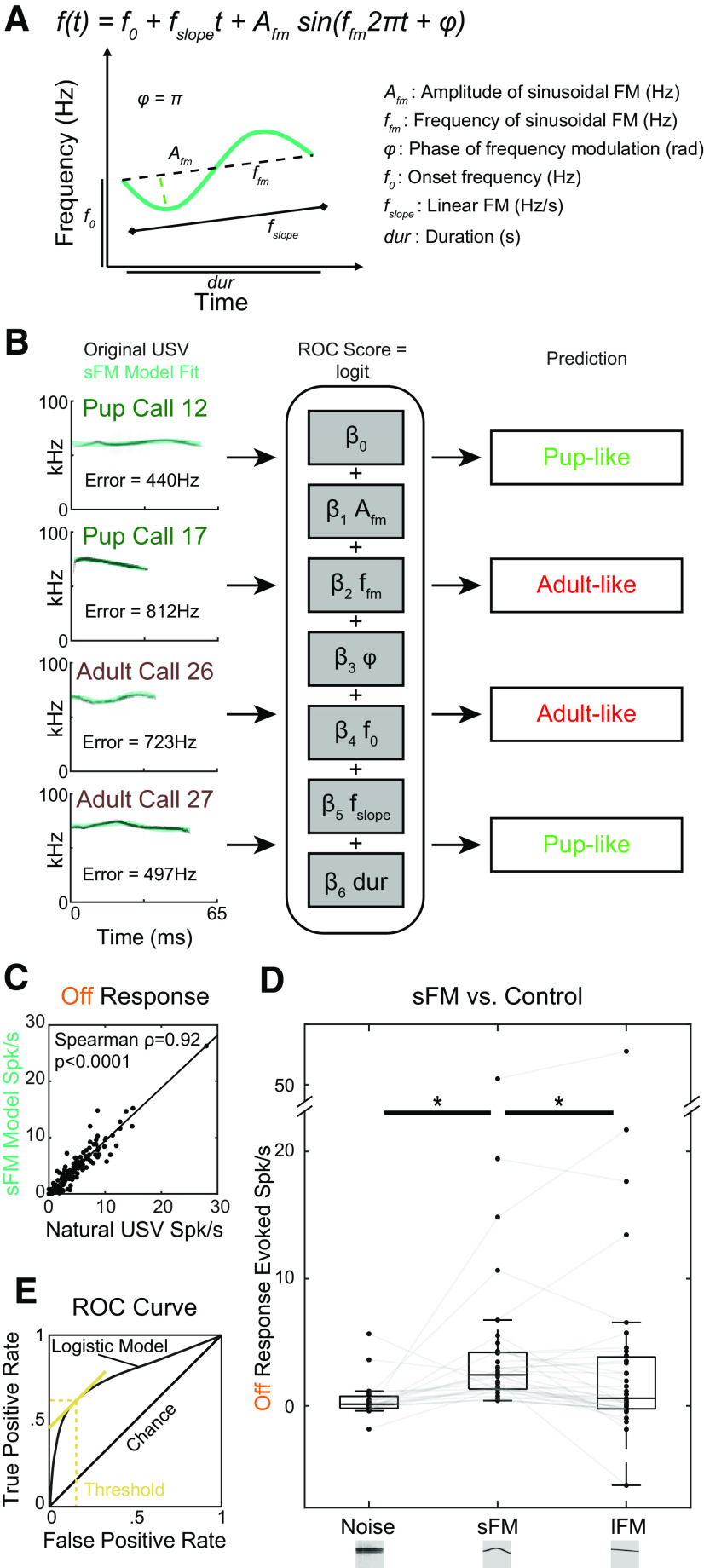
Combined sinusoidal and linear FM models of USV trajectories can discriminate call categories. ***A***, Schematic illustrating the sFM model equation. ***B***, sFM fits to 4 different USVs, which are then classified based on the sFM parameters by nominal logistic regression modeling. Left, Frequency trajectories of sFM models (light green) of ground-truth pup and adult USVs, overlaid on the spectrograms of the original USVs (grayscale). For each call, a set of 6 parameters was generated. These parameters were then used to predict the call's likely category (pup-like, light green; adult-like, red) using a nominal logistic regression model. Error was defined as the Sqrt(sum squared error/number of samples). Model parameters: β_Afm_ = −1.6e-5; β_Ffm_ = −2.4e-3; β_φ_ = 8.0e-2; β_f0_ = −9.4e-6; β_fslope_ = −2.1e-8; β_dur_ = 3.4e-2; Model χ^2^ = 7.2e3, *p* < 0.0001. ***C***, Comparison of Off response spike rates evoked by an original, natural USV versus its sFM model. There is a high correlation between responses (Spearman ρ = 0.92, *p* < 0.0001). ***D***, Control stimulus set comparing Off responses to sFM models of USVs versus just the lFM component of the model, and the spectrally matched noise (right, for sFM vs lFM: n_stim*su_ = 35, n_su_ = 20, n_anim_ = 13; for sFM vs Noise: n_stim*su_ = 19, n_su_ = 14, n_anim_ = 8). *p < 0.01 (Paired Wilcoxon Signed Rank). ***E***, Performance of the nominal logistic regression model according to an ROC analysis in correctly classifying ground-truth pup calls as pup-like (true positive) and in incorrectly classifying ground-truth adult calls as pup-like (false positive). Threshold (gold) represents the cutoff at which the true positive rate is maximized and the false positive rate is minimized. The model performs above chance (AUC = 0.76, *p* < 0.0001 via Boostrap analysis, *N* = 1000).

**Figure 3.**
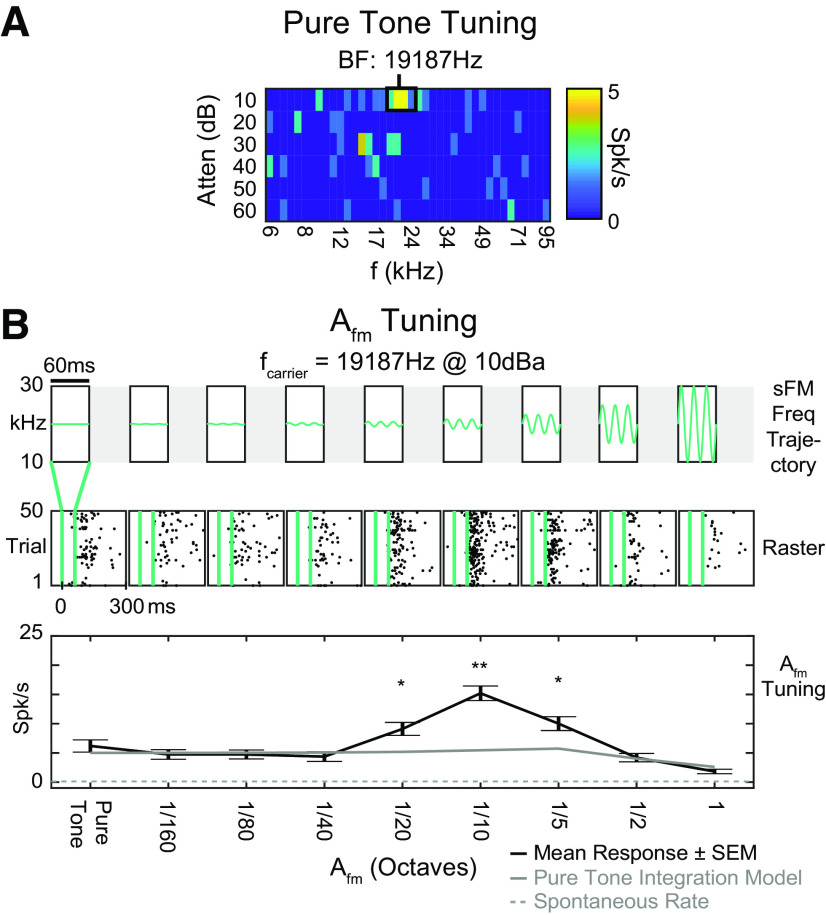
Auditory cortical SUs can be tuned to A_fm_ in sFM sounds varied around BF. ***A***, Pure tone tuning response area (SU 2981). The SU's absolute spike rate is represented by the heat map color, with hotter colors representing higher spike rate. Red box represents the SU's best responding area, with a BF of 19,187 Hz. ***B***, A_fm_ tuning to 60 ms sFM varied around this SU's BF. Top, Schematic frequency trajectories of each stimulus, with all other parameters fixed at: f_fm_ = 50 Hz, φ = 0, f_0_ = BF (19,187 Hz), f_slope_ = 0 Hz/s, dur = 60ms. A_fm_ is varied in logarithmic steps from 0-1 octave. Middle, Raster responses to stimuli delivered within the vertical blue lines. Black dots represent individual spikes. Bottom, Mean response tuning curve (black). Error bars indicate SEM. Spontaneous rate (dotted gray line) and rate predicted from integrating the pure tone (PT) tuning curve (green) are also shown. *p < 0.01; **p < 0.0001; Bonferroni-corrected *t* test.

**Figure 4.**
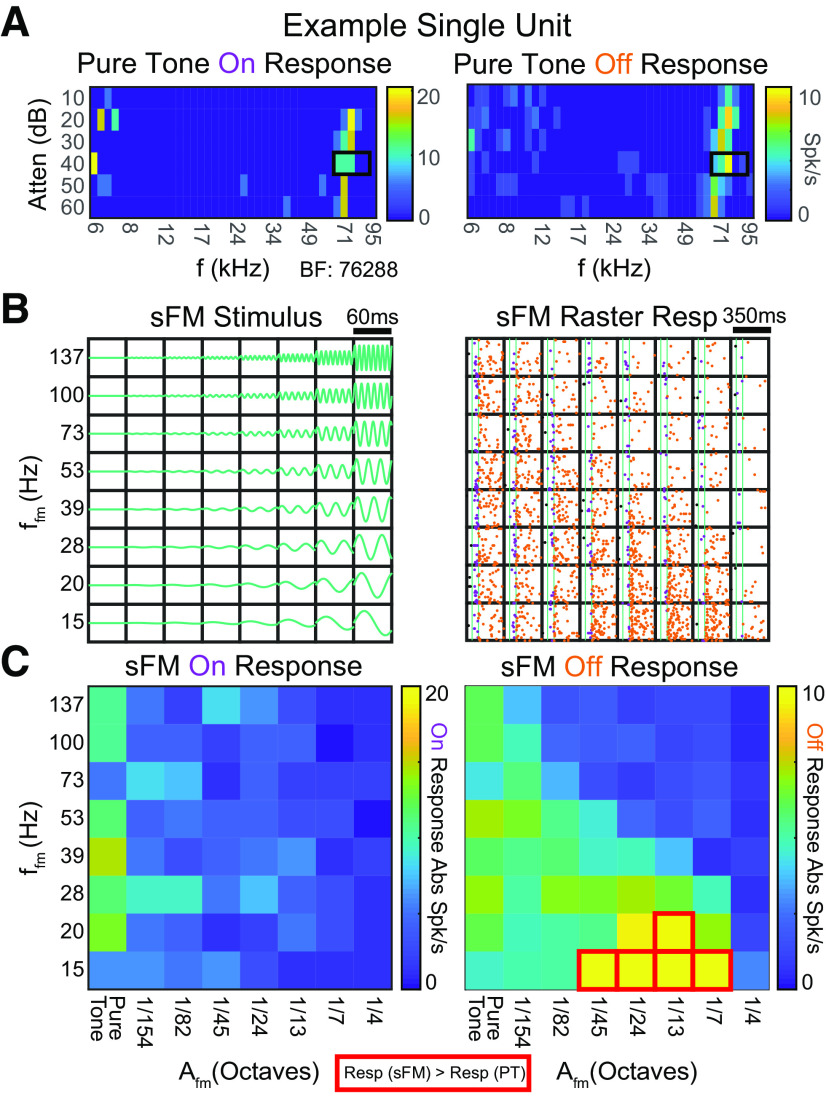
Auditory cortical SUs can be tuned to combinations of A_fm_ and f_fm_ in sFM sounds varied around BF. ***A***, Pure tone tuning response areas for the On response (left) and Off response (right) of SU 3009. The SU's absolute spike rate is represented by the heat map color. Red boxes represent the SU's best responding areas for their On and Off responses, with a BF of 76,288 Hz. ***B***, Left, Schematic frequency trajectories of the 60 ms duration A_fm_ × f_fm_ tuning stimulus. Right, Raster responses of the SU, with purple dots representing individual spikes falling within the On response window and orange dots representing those in the Off response window. ***C***, Heat map representing the firing rate of the SU within the On response window (left) and Off response window (right). In each grid, the left-most column, where A_fm_ is 0, consists of 8 sets of pure tone trials (25 trials each), and thus reflects the inherent variability in responses to the identical pure tone (PT) sound. Red boxes outline those stimuli that evoked a significantly higher spike rate compared with the PT spike rate (*p* < 0.05, Bonferroni-corrected Wilcoxon Rank Sum).

**Figure 5.**
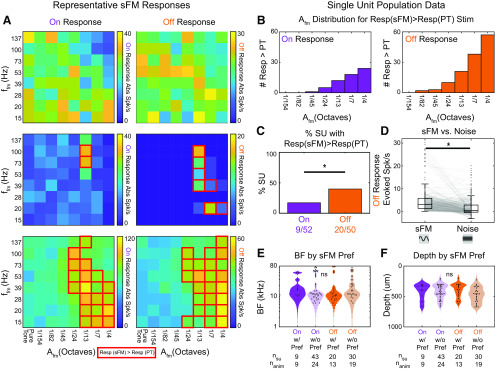
sFM tuning is seen across the population of auditory cortical neurons. ***A***, On and Off response heat maps for A_fm_ × f_fm_ stimuli in the case of three other example SUs: top, SU 3164; middle, SU 3064; bottom, SU 3010. Red boxes same as in Figure 4*C*. ***B***, Histogram of responses significantly modulated by subtle sFM. Each panel shows the distribution of A_fm_ values across SUs and A_fm_ × f_fm_ combinations for sFM stimuli that elicited a significantly greater response than the SU's pure tone response for On responses (top) and Off responses (bottom). Significance was based on each SU's statistical testing (see red boxes in Fig. 4*C*). ***C***, Proportion of SUs whose On or Off responses are significantly modulated by sFM trajectories, compared with a pure tone. The On response proportion was significantly lower than the Off response proportion (*p* = 0.023, Fisher's exact test). ***D***, The sFM trajectory elicits stronger Off responses than noise bursts with matched bandwidth (left, n_stim*su_ = 280, n_su_ = 40, n_anim_ = 21). *p < 0.05 (Paired Wilcoxon Signed Rank). ***E***, SU BF distributions did not differ depending on whether the SU showed a (non-PT) sFM preference or not in their On or Off response (not significant, Kruskal–Wallis). ***F***, SU depth distribution did not differ depending on whether the SU showed a (non-PT) sFM preference or not in their On or Off response (not significant, Kruskal–Wallis). ns: not significant.

**Figure 6.**
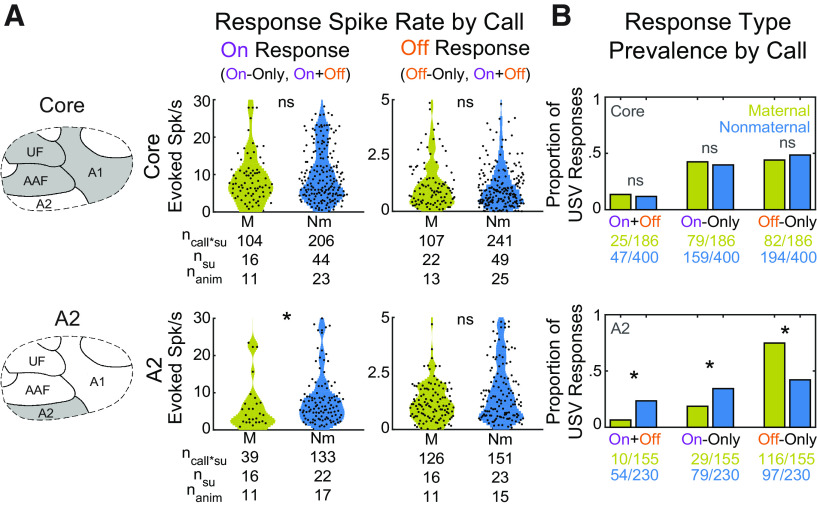
On and Off responses to USVs differ across field and maternal experience. ***A***, Response spike rates on a per-call basis divided by auditory cortical field [core (left, top row) and A2 (left, bottom row)] and by On (middle) and Off (right) Resp. Evoked spike rates are calculated by subtracting an SU's spontaneous rate. Mustard represents SUs from maternal animals. Blue represents SUs from Nm animals. *p < 0.0001 (Bonferroni-corrected Wilcoxon Rank Sum). ***B***, Prevalence of each type of response on a per-call basis, divided by auditory field [core (top) and A2 (bottom)]. *p < 0.0001 (Fisher's exact test). ns: not significant.

**Figure 7.**
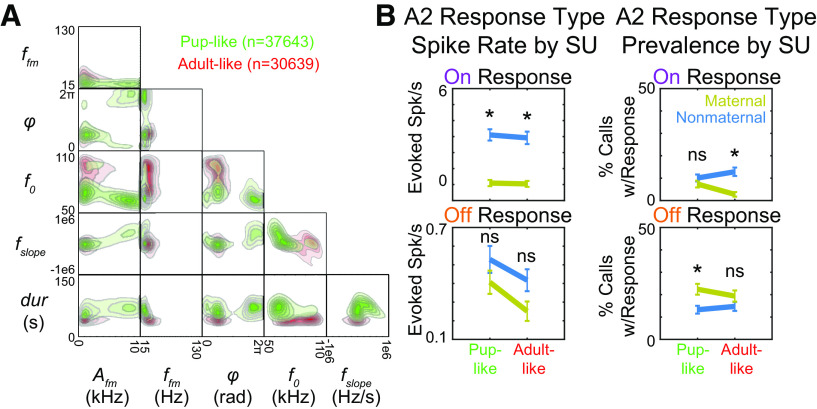
Maternal plasticity in A2 responses to USVs depends on whether USV trajectories are pup-like or adult-like. ***A***, Distribution of the six sFM parameters for pup-like (blue) and adult-like (red) USVs. Separation between categories is apparent for A_fm_, duration, phase (φ), and onset frequency (f_0_). ***B***, SU spike rate responses (left) and prevalence (right) to our curated (medium and long duration) USVs during the On response window (top) and Off response window (bottom), separated by USVs that are pup-like versus adult-like, as classified by the logistic regression model. *p < 0.05: Bonferroni-corrected Wilcoxon Rank Sum (spike rate responses) or Fisher's exact test (prevalence). ns: not significant.

**Figure 8.**
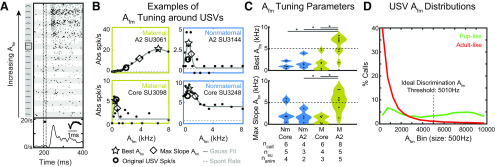
A_fm_ tuning around USVs changes in A2 with experience. ***A***, Raster responses to sFM models with A_fm_ varying around an example SU's best call (boxed spectrogram in left column). Tuning stimuli, presented during the period indicated by the red lines, include 19 different sFM models with A_fm_ varying from 0 to 8000 Hz, which spans the range of natural USV A_fm_ values. The other sFM parameters are fixed at values matching the best USV. PSTH shows pooled spike rate responses to all stimuli, highlighting the responses after the Offset of the sounds. ***B***, Example A_fm_ tuning curves of four different SUs, based on the full responses. Two SUs from A2 (top) and two from core (bottom) are shown from both maternal (left) and Nm (right) mice. Responses are fit to a Gaussian, whose peak is represented by a red circle, and point of maximum slope by a blue circle. Black circle represents spike rate in response to the original USV. Dotted gray line indicates spontaneous rates. ***C***, Best A_fm_ (top) and maximum slope A_fm_ (bottom) values according to animal experience group (M, Maternal) and auditory field. *p < 0.05 (Tukey Kramer's HSD). ***D***, Histogram of A_fm_ in either the pup-like (blue) or adult-like (red) USVs, as a proportion of the calls in each respective category. The ideal observer discrimination threshold for pup-like versus adult-like calls, as determined by ROC, is indicated by the black line at A_fm_ = 5010 Hz.

The electrophysiological studies reported here compare SUs recorded from core auditory cortex to A2, and between these fields in maternal and nonmaternal mice. Individual SUs were characterized in detail for their responses to natural vocalizations, pure tones, and frequency modulated stimuli, with Figures 1, 3, 4, and 8 showing example SUs. We tested frequency modulated stimuli that were either varied parametrically around the BF of the recorded SU and/or around individual USVs. Population analyses comparing fields and/or animal groups are shown in Figures 2, 5–8. Statistical analyses were performed in MATLAB (The MathWorks) and JMP Pro 13 (SAS). As detailed in each of the subsequent Data analysis sections below, statistical tests were often based on contingency tables for which we applied Fisher's exact test. For other comparisons, we used the Wilcoxon Kruskal–Wallis, Rank Sum (unpaired) or Signed Rank (paired) tests for nonparametric data. Figure 8 applied ANOVA (with *post hoc* Tukey Kramer's Honestly Significant Difference [HSD]), since the data could not be distinguished from a normal distribution (MATLAB lillietest). For multiple comparisons, we applied Bonferroni correction unless otherwise noted. Data samples are reported on a per call, per SU, and per animal basis in the text, figures, or figure legends. Test statistics and exact *p* values are given in the text or figure legends.

### FM tuning around pure tone BFs

#### Experimental details

##### Animal groups

In addition to using intact female mice of the strain and age described in the overall methods, a subset of animals used for sFM tuning around BF were also part of a separate pilot study and had been ovariectomized and given either systemic β-estradiol or oil vehicle subcutaneous implants. The SUs from these animals did not differ significantly in standard measures of neural responses (Table 1). While removing these animals did not change the overall results found, they were included in this section of the analysis for greater statistical power, and to illustrate the ubiquity of neural tuning to short FMs around the BF.

**Table 1.** No significant differences between SU from OVX and non-OVX animals

Measure (mean ± SD)	OVX (*n* = 13 SU, 7 mice)	No OVX (*n* = 48 SU, 24 mice)	*p*
Spontaneous rate (spk/s)	3.36 ± 5.1	1.18 ± 1.386	0.15
BF (kHz)	24.2 ± 21.8	23.1 ± 21	0.88

##### Sound stimulus playback

Pure tone frequency tuning curves were first obtained with 60 ms duration tone pips (10 ms cos^2^ ramp) at 6 sound levels (15–65 dB SPL) and 30 frequencies (log-spaced 5-80 kHz), repeated 15 times each and presented in pseudorandom order. To assess A_fm_ tuning, 60 ms duration sFM stimuli with f_0_ equal to the BF (linear FM f_slope_ = 0, φ = 0) were then played back with 9 steps of increasing A_fm_ (octaves: 0, 1/160, 1/80, 1/40, 1/20, 1/10, 1/5, 1/2, 1). Each sFM sound, presented at the best pure tone level, was repeated 50 times in randomly interleaved trials. To assess combined A_fm_ and FM frequency (f_fm_) tuning, 60 ms duration sFM stimuli with f_0_ equal to the BF (f_slope_ = 0, φ = 0) were presented. f_fm_ was varied in 8 logarithmic steps from 15 to 137 Hz, while A_fm_ was varied logarithmically from 0-1/4 octave (0, 1/154, 1/82, 1/45, 1/24, 1/13, 1/7, 1/4). A total of 25 randomly interleaved trials per stimulus (presented at the best pure tone level) were collected. For each of these stimuli, we also presented a corresponding bandwidth-matched noise stimulus of the same duration, with noise generated in real time with RPvdsEx in Brainware (Tucker Davis Technologies). The amplitude of the noise was scaled to match the RMS of the corresponding sFM stimulus. A total of 280 pairs of sFM and matched noise stimuli were played to *n* = 40 SUs in *n* = 21 animals. An additional linear FM (lFM) control was used, where an sFM model of a mouse USV was paired with only the linear component of the same sFM model. A total of 35 pairs of sFM and lFM were played to *n* = 20 SUs in *n* = 13 animals.

For all stimuli described, each trial lasted 600 ms, with stimulus playback beginning at 200 ms during a trial, resulting in an interstimulus interval of ∼540 ms for 60 ms duration sounds (with some trial onset jitter due to Brainware's trial-based system).

#### Data analysis

##### Pure tone integration model for prediction of A_fm_ spike rates

In order to predict expected spike rates for A_fm_ stimuli from a SU's pure tone tuning, we computed the power spectral density (MATLAB, pwelch.m) of each of the A_fm_ stimuli. The amount of power in each frequency bin of the power spectrum was multiplied by corresponding frequency bin's spike rate in the SU's pure tone tuning curve, normalized such that the evoked response from the BF pure tone for both the A_fm_ tuning stimulus (A_fm_ = 0) and the pure tone frequency tuning curves were matched. As the number of frequencies played back during pure tone tuning is fewer than the number of (positive) frequency bins in the power spectrum (*n* = 129), the pure tone tuning curve was interpolated linearly across missing frequency bins.

##### Analysis of A_fm_ and f_fm_ tuning around BF

We analyzed the 8 × 8 A_fm_ and f_fm_ tuning stimulus responses to test whether SUs show preference for sFM stimuli over a pure tone. Data used in this analysis included *n* = 61 SUs recorded from *n* = 31 animals. SU responses were divided into the On response (during 60 ms window of stimulus playback) and the Off response (window defined starting after stimulus ends until the response drops back to spontaneous levels). *n* = 52 SUs exhibited an On response, and *n* = 50 exhibited an Off response. Absolute spike rates were calculated over the On and Off window for each stimulus. To determine whether a response to a specific combination of A_fm_ and f_fm_ significantly differed from that for its BF pure tone, the collection of spike rates from all trials of a given A_fm_ and f_fm_ combination was compared with the collection of spike rates from all pure tone trials using the nonparametric Wilcoxon Rank Sum test. To account for multiple testing, the Bonferroni correction was applied, with the α level taken as (0.05/64) or 0.00078. An SU was considered to prefer sFM over pure tone if there was at least one set of sFM parameters (where A_fm_ was non-zero) with significantly greater response than the BF pure tone for either its On or Off response. Comparisons of the number of On versus Off responses that had a significantly greater response to at least one sFM stimulus compared with pure tone was conducted via Fisher's exact test.

##### sFM matched noise and linear FM analysis

For comparison of responses between sFM and matched-bandwidth noise stimuli, evoked responses were calculated during the On, Off, and the entire evoked response windows, with the spontaneous rate (as defined by the spike rate during a 200 ms prestimulus silent period) subtracted. A nonparametric Paired Wilcoxon Signed Rank Test was performed comparing the sFM and its paired noise-evoked spike rate. In this analysis, a total of *n* = 280 paired sFM and noise stimuli were played to *n* = 40 SUs in *n* = 21 animals. For comparing between sFM and lFM, we also calculated the responses during the On, Off, and entire evoked response time windows, normalizing by subtracting the spontaneous rate, followed by performing the Paired Wilcoxon Signed Rank Test. For the linear FM control, a total of *n* = 35 paired sFM and lFM stimuli were played to *n* = 20 SUs from *n* = 13 animals. As a prerequisite to be included for this analysis, all SUs used here showed an excited response to the stimuli, as judged by 2 independent scorers (disagreements, which happened <5% of the time for call-excited [Exc] responses, were not included in the analysis).

### USV frequency trajectory tuning plasticity

#### Experimental details

##### Animal groups

Mice had varying levels of pup experience. The Nm animal group consisted of pup-naive females, pup-naive females that have only been passively exposed to pup USVs without social interaction, and females that had previously acted as cocarers with a mother, but at the late postweaning time point (>P21) for electrophysiology. At this time point, pup calls were no longer salient, based on the lack of preferential phonotaxis to pup USVs (Lin et al., 2013). The maternal animal group consisted of postweaning primiparous mothers (P21, which still find the pup USVs salient) (Lin et al., 2013), as well as cocarers who were just caring for a litter of pups up to P5–P7 before electrophysiology. For all mice in the maternal group, pup retrieval was conducted on postnatal days P5–P7, in which pups were scattered in the home cage and animals were given 5 min to retrieve pups back to the nest. Pup scattering was repeated for a total of 3 times. Only animals that performed pup retrieval successfully were included in the maternal animal group. No hormonally manipulated animals were included in this section.

##### sFM modeling of USVs

Mouse pup and adult USVs are complex, single-frequency whistles that have naturally variable frequency trajectories. In order to capture and parameterize the various frequency trajectories of mouse USVs, we used a parameterized sinusoidal plus linear frequency-modulated tone model (sFM) to fit to the frequency trajectory of each call (see Fig. 2*A*). The parameterized model contains a total of 6 parameters: duration (dur), onset frequency (f_0_), sFM amplitude (A_fm_), sFM frequency (f_fm_), sFM phase (φ), and linear FM slope (f_slope_). Parameters were fit to minimize the mean squared error between the model and call [reported error = Sqrt(sum squared error/number of samples)]. For playback during neural recording, we used a curated natural USV stimulus set containing 18 pup and 18 adult, ground-truth USVs that were matched for duration, onset frequency, and degree of FM at onset (Shepard et al., 2015). In synthesizing the sounds, we applied the original amplitude modulation of the natural USV.

##### Sound stimulus playback

USVs (*n* = 36) plus a silent stimulus were played to animals, with up to 50 trials per stimulus, randomly interleaved, as described previously (Shepard et al., 2015). For a subset of SUs, we also played back the original set of 36 USVs plus 36 sFM models of each USV, randomly interleaved for a total of 25 trials per stimulus.

For each neuron that showed an evoked response to USVs, we then presented additional sFM stimuli optimized around the call that elicited the best response, to assess tuning for sFM parameters. This stimulus contained sFM exemplars with f_0_, f_slope_, f_fm_, φ, and dur equal to that of the call eliciting the best response, while A_fm_ was varied in 19 logarithmic steps across the range of natural A_fm_ values in USVs (A_fm_ = 180.78 × exp(0.1051 × *n*); *n* = [2:2:38]). The original best-response call's A_fm_ was also included, for a total of 20 different randomly interleaved stimuli in that set, repeated a total of 30 trials per stimulus. If a SU responded to both pup and adult USVs, two A_fm_ tuning stimuli were presented: with one centered around the best pup USV and the other around the best adult USV. In some instances, we also played back a control stimulus set with noise spectrally matched to each of the 20 A_fm_ tuning stimuli. Within the spectrally matched noise control stimulus, for each corresponding A_fm_ tuning stimulus (*n* = 20), three instances of randomly generated white noise, each with different random seeding, were first generated and then filtered based on the spectral content of the original A_fm_ stimulus, for a total of 60 stimuli. Each of the 60 stimuli was played for a total of 10 trials per stimulus, such that total presentation time approximately matched that of the sFM-only A_fm_ tuning stimulus. Our ability to hold SUs over the course of this protocol varied from site to site, so not all stimuli were played to all units.

#### Data analysis

##### Spike density plot

We generated a spike density plot (MATLAB, dscatter.m, freely available online) (Eilers and Goeman, 2004) to visualize the overall population spiking activity across the *n* = 36 natural mouse USVs, sorted by increasing USV duration (see Fig. 1*A*). Default smoothing settings were used, in which smoothing was conducted over 20 bins, with the time axis divided into 0.1 ms bins (6000 bins across a 600 ms period). For visualization purposes, the *y* axis is divided into 100 bins per stimulus (3600 bins for 36 stimuli), and randomized jitter ([0–1 bins]) was applied to each individual spike along the *y* axis. A total of *n* = 136 Exc SUs were included, pooling across region and animal group.

##### On/Off prevalence and spike rate analysis

In analyzing On and Off responses to USVs on a per-call and per-SU basis, the 12 shortest duration calls in the stimulus library were excluded (∼12-15 ms duration calls with faded numbers; see Fig. 1*A*, bottom row), as On and Off responses to these short calls could not be definitively differentiated. On and Off responses to individual calls were classified by 2 independent investigators (K.K.C. and D.B.K.); spiking above spontaneous levels (measured over the 200 ms prestimulus period) during the call's playback signified an On response, whereas spiking above spontaneous levels during a 300 ms window after the call ended signified an Off response. When both On and Off responses were noted for the same call, that response was deemed On+Off. Such responses could either arise from distinct On and Off components separated by minimal firing or from delayed On firing that was sustained into the Off period; these were not differentiated. Data used in this analysis came from *n* = 3264 call responses (*n* = 971 responses were Exc) played to *n* = 136 Exc SUs from *n* = 55 animals. We also considered and implemented an automated algorithm to determine when there was a spike rate increase at least 2 SDs above the spontaneous rate. However, although there was good agreement for most cases, it misclassified cases when SUs had very low spontaneous activity, or diffuse responses, or very brief responses. Thus, manual scoring was considered a reasonable and more reliable alternative.

For analyses on a per-SU basis, SUs were classified as On-Only, Off-Only, or On+Off SUs based on their Exc responses to the 24 longer-duration calls. On and Off responses of an SU did not necessarily have to be from the same call for that SU to be classified as On+Off.

##### Logistic regression modeling for classification of sFM parameter combinations as pup-typical versus pup-nontypical

A nominal logistic regression was performed using our library of 10,353 adult and 57,989 pup ultrasonic calls, where calls <4 ms duration and minimum frequencies <45 kHz were excluded. The nominal logistic regression model was fit using the six sFM parameters (A_fm_, f_fm_, φ, f_0_, f_slope_, dur) to best predict “pup” (pup-like) and “adult” (adult-like) labels. The model followed the format: Score = logit(β_0_ + β_1_ A_fm_ + β_2_ f_fm_ + β_3_ φ + β_4_ f_0_ + β_5_ f_slope_ + β_6_ dur), where each of six β coefficients were fit to maximize prediction accuracy. An ROC curve was constructed using the resulting model, and a threshold score was selected that maximized the sensitivity and minimized 1 – specificity. Significance of the ROC was assessed using a bootstrap analysis (*N* = 1000).

##### A_fm_ tuning analysis

A Gaussian fit ($a\cdot e^{\left(-{\left(\frac{x-b}{c}\right)}^{2}+d\right)}$) was applied (MATLAB fit.m) to the spike rate as a function of A_fm_ to determine the peak (Gaussian mean, *b*) and bandwidth (Gaussian SD, *c*) of an SU's A_fm_ tuning. Fit parameter initial values and [lower bound, upper bound] were as follows: a = 1 [0, Infinity], b = empirical best A_fm_ (i.e., gives the max spike rate) [0, 10,000], c = empirical A_fm_ half-maximum to half-maximum width [0, 10,000], d = 0 [0, Infinity]. Reported best A_fm_ values were taken from the Gaussian fit tuning curves. When using the A_fm_ corresponding with the maximum measured spike rate instead of a Gaussian fit, the results remained the same as reported, but the fit provided a way to estimate the point of maximum slope in the tuning curve (i.e., the SD of the Gaussian).

Results were divided by animal experience group (maternal or nonmaternal) as well as auditory cortical region (core or A2), and group comparisons were conducted with a nonparametric Wilcoxon Kruskal–Wallis test followed by the *post hoc* Tukey Kramer HSD method, with *p* < 0.05 taken as the significance level. A_fm_ tuning around USV parameters was measured for *n* = 25 calls played to *n* = 18 SUs from *n* = 15 animals. When conducting analysis by call, by SU, or by animal, results remained significant.

## Results

### Neurons show heterogeneity in timing of responses to USVs

Adult female mice come to recognize USVs emitted by isolated pups after maternal experience, as demonstrated by their preference to approach a pup USV over a neutral sound (Ehret et al., 1987; Lin et al., 2013). Adult males emit another category of USVs that is also meaningful for females, and these overlap the pup USV frequency range, although auditory cortical SUs in maternally experienced females can differentiate these (Shepard et al., 2015). We used a stimulus set of *n* = 36 curated, natural pup, and adult USVs with matched onset frequency and duration properties (Fig. 1*A*) to characterize the sensitivity of SUs in both core and secondary (A2) auditory cortex to the frequency trajectories of these short sounds. Following previous work (Galindo-Leon et al., 2009; Shepard et al., 2015), we classified SUs from maternal and nonmaternal animals (see Materials and Methods) as Exc, call-inhibited (Inhib), or call-nonresponsive (NR). Notably, a sizable fraction of neurons we recorded were not responsive to any USVs (NR, *n* = 449 of 720; Fig. 1*B*), evidencing our attempts to reduce unintended recording bias against those SUs that have lower spontaneous firing rates or highly selective responses. SUs that were purely Inhib have been studied previously (Galindo-Leon et al., 2009; Lin et al., 2013), wherein we found that maternal experience led to a stronger call-evoked suppression of firing in regions of core auditory cortex tuned below the ultrasonic call frequency range, consistent with changes in cortical inhibition found in mothers (Cohen et al., 2011; Krishnan et al., 2017). This is believed to improve the population level neural contrast between neurons excited by pup USVs from those that are not (Banerjee and Liu, 2013). We did not consider such responses further here.

Exc responses (*n* = 136) across core and A2 were pooled to create a spike density plot for USVs to visualize general patterns of firing. The population response revealed prominent neural activity both while the stimulus was On for most USVs, as well as in the Off portion after the end of a USV, especially for the longest USV durations (Fig. 1*C*). We classified each Exc SU into one of three subcategories based on their response to the longer-duration calls, where On and Off components could be more clearly differentiated. “On-Only” SUs had an On response to at least 1 of 24 longer-duration calls calls, but no Off responses to any (Fig. 1*D*). On+Off SUs had an On response to at least 1 of 24 calls, and an Off response to at least one call as well (Fig. 1*E*); this could include On+Off responses for the same call. Finally, Off-Only SUs had an Off response to at least 1 of 24 calls, but no On responses to any (Fig. 1*F*). Most Exc SUs we recorded were On-Only, but ∼44% of Exc SUs had some form of Off response, falling into either the On+Off or Off-Only SU groups. Hence, although there was considerable heterogeneity across auditory cortical SUs in when they increased their firing rate in response to USVs, nearly half did so after the end of at least one of the calls.

These late responses were particularly interesting because their timing could allow for a sensitivity to sound features beyond the onset, facilitating the discrimination of different calls. For example, in the case of the SU shown in Figure 1*F*, although calls 11 and 2 were matched in their onset frequencies and durations, the former drives a strong Off response, whereas the latter does not. Such selective responses hint that individual neurons can be sensitive not only to the static spectrum of a short sound, but also to how its frequency trajectory changes over time. To test this for the coding of USVs, we next sought to model the frequency trajectories of natural USVs so that we could manipulate their acoustic parameters.

### Combined sinusoidal and linear FM models of USV trajectories can discriminate call categories

We fit a large library (Liu et al., 2003) of 57,929 pup USVs and 10,353 adult USVs to a 6-parameter sFM model that added linear to sinusoidal modulation to accurately describe the frequency trajectories of USVs (Fig. 2*A*; see Materials and Methods). The mean square error for the fit to each call was relatively small, averaging 639 Hz, compared with the 60-80 kHz range of the USVs themselves (Fig. 2*B*, left). Both Off responses and full responses (data not shown) elicited by these sFM model USVs were highly correlated with responses evoked by their paired, natural USVs with the same amplitude envelope (Fig. 2*C*; Spearman ρ = 0.92, *p* = 1.02 × 10^−60^, *n* = 146). Hence, our parameterized FM largely captured the frequency trajectory features in USVs that drive SU firing.

We then found that the specific time-dependent trajectories of the natural USVs were important for producing the observed neural responses. In a subset of SUs, we presented not only our full sFM model of a USV in our library, but also a narrow-band noise model whose power spectrum was exactly matched to that of the calls (see Materials and Methods) and/or just the lFM component of our sFM model. The latter lFM control addressed the possibility that firing to spectrally matched noise was simply suppressed by the noise spectrum engaging a SU's inhibitory sidebands (Young and Brownell, 1976). In all cases, we applied the amplitude envelope of the paired natural call. We found that Off responses for our full sFM+lFM model were significantly stronger across our SU population compared with both the spectrally matched noise (which was not significantly different from spontaneous firing, one-sample Wilcoxon, *p* = 0.12) and the lFM component alone (Fig. 2*D*; Paired Wilcoxon Signed Rank: vs noise, W = 22, *Z* = −2.94, *p* = 0.003; vs lFM, W = 478, *Z* = 2.67, *p* = 0.008). Hence, late SU firing after the end of a USV was indeed sensitive to how these short USVs changed over time from one frequency to another, with the natural trajectory eliciting better responses.

### Off responses to generic sFM sounds are sensitive to time-dependent frequency trajectories

Given the observed preference for the trajectory of natural USVs, we wondered whether the sensitivity to time-dependent frequency trajectories was specific for these vocalizations, or if they might be found more generally for variations in the trajectory of other short sounds. We explored this by using 60-ms-long tones (comparable to the duration of pup USVs; Fig. 1*A*) with sFM variations tailored around a SU's pure tone BF. For the example SU in Figure 3*A*, frequency excursions (A_fm_) smaller than the typical spectral width of pure tone tuning curves (Schreiner and Sutter, 1992; Linden et al., 2003; Joachimsthaler et al., 2014) drove better responses than the constant pure tone BF itself (Fig. 3*B*). This SU had a peak in A_fm_ tuning at 1/10 octave (temporal modulation frequency f_fm_ = 50 Hz), with an evoked spike rate more than twice that predicted from just integrating the pure tone excitatory tuning curve over the same spectral range (Fig. 3*B*, green line). Larger A_fm_ values reduced firing rates from the peak, which would not be explainable just by its excitatory sensitivity to the brief sound's static spectrum.

Varying the sFM's temporal modulation frequency (f_fm_) in addition to A_fm_ gave us further insight into whether SUs might be sensitive to how a short sound's frequency content unfolds in time. Looking separately at response components during (On) and after (Off) the sFM sound for a different example SU (Fig. 4*A*), we saw that its Off response firing significantly increased as the tone's frequency crossed the same A_fm_ = 1/13 octave range at slower f_fm_ rates (Fig. 4*B*), an effect that also held at neighboring A_fm_ values. However, tuning was different for On and Off response components (Fig. 4*C*), despite being similar for static pure tones (Fig. 4*A*). Since the frequency trajectory immediately preceding the On and Off response differs at the start and end of a modulated sFM sound but is the same for a static tone, On versus Off response tuning differences are consistent with a sensitivity to how the frequency trajectory changes in time.

### Neurons sensitive to sinusoidal frequency trajectories are found across the hearing range and across cortical depths

We assessed the On and Off response to sFM around BF across a population of *n* = 61 core (A1, AAF, ultrasound field, *n* = 44) and secondary (A2, *n* = 17) auditory cortical SUs. Of these, *n* = 52 had an On response to at least one of these stimuli, and *n* = 50 had an Off response. SUs responded in varied ways in both their On and Off responses (Fig. 5*A*). For some SUs, sFM sounds elicited spike rates (either On or Off) that were comparable to pure tone responses (Fig. 5*A*, top row) for all the stimuli within our sFM parameter space; these were considered nontuned for sFM around BF. Many SUs, however, exhibited strong preferences either for sFM within narrow regions of combined, non-zero A_fm_ and f_fm_ (Fig. 5*A*, middle row; red lines outline sFM stimuli that elicited a response significantly larger than for pure tones), or for larger ranges of A_fm_ and/or f_fm_ (Fig. 5*A*, bottom row). Only 7 of 61 SUs (3 of 52 On, 4 of 50 Off) showed significantly suppressed spiking for one of the sFM stimuli compared with pure tones, and thus were not considered in detail further.

Excitatory preferences for sFM over constant pure tones could be seen in an SU's On and/or Off response. While larger FMs typically elicited more responses that were significantly higher than pure tone responses, A_fm_ as low as 1/24th of an octave (and on rare occasions even less) could modulate spiking in both the On and Off responses (Fig. 5*B*). To determine which response type was more sensitive to sFM, we measured how many of the SUs with either an On or Off response had at least one sFM stimulus that elicited a significantly greater spike rate than for the BF pure tone (*p* < 0.05, Bonferroni-corrected Wilcoxon Rank Sum). We found that Off responses were significantly more likely than On responses to have a preferred sFM different from pure tone (40% vs 17%, Fisher's Exact, *p* = 0.023; Fig. 5*C*), as might be expected if an SU were responding to a sound's frequency trajectory and not just its spectrum. Indeed, even for those SUs with a sFM-preferring On response, those On responses were typically more sustained, tonic, or late-onset. Hence, more of a sound's history than just its onset could be integrated to affect the neural response, as would be the case for Off responses.

To further test neural sensitivity to short frequency trajectories as they unfold over time, and not just a stimulus' static spectrum, we recorded responses of SUs during playback of a narrowband noise that was bandwidth-matched to each of the sFM stimuli in our 8 × 8 A_fm_ × f_fm_ grid. SU Off responses were significantly higher for sFM compared with their matched noise (Fig. 5*D*, left; Paired Wilcoxon Signed Rank, W = 28,070, *Z* = 6.19, *p* = 5.87 × 10^−10^). Hence, these data suggest that Off responses are particularly sensitive to frequency trajectory variations present in short sFM stimuli.

SUs that showed a preference for sFM over pure tones had BFs varying across the entire mouse hearing range from 6 to 80 kHz, with BF distributions that were not significantly different between On or Off responses (Fig. 5*E*). These BF distributions were also comparable to those seen for SUs without a preference for sFM. The SUs with BFs >50 kHz correspond to those found in either the ultrasound field of the core or from A2, both of which can have higher frequency BFs (Stiebler et al., 1997; Joachimsthaler et al., 2014; Shepard et al., 2015). Finally, the distribution of cortical depths between SUs with or without sFM preference also did not differ (Fig. 5*F*); SUs showing preference for sFM spanned the entire range of cortical depths that were sampled (200–700 µm).

Together, these results suggest a general sensitivity of auditory cortical neuronal firing rates to parameters that delineate the frequency trajectories of arbitrary sounds across the mouse's hearing range, especially in their Off responses. Presumably such sensitivity to subtle FM would be useful in representing features of acoustic categories that must be behaviorally discriminated, even when those sounds overlap in their overall static spectrum. If so, we would expect that experiences to make a sound category meaningful could drive neural plasticity that would be reflected in the tuning to such modulations. We addressed that possibility next by investigating whether and where neural plasticity in USV trajectory sensitivity emerges after the behavioral relevance of a specific USV category is acquired through experience.

### Strength of On responses to USVs decreases with experience

In numerous paradigms, behaviorally meaningful experience learning about sounds induces different forms of auditory cortical plasticity (Recanzone et al., 1993; Liu et al., 2006; Pienkowski and Eggermont, 2011). To determine whether maternal experience caring for pups might alter tuning for natural USV frequency trajectories, we analyzed whether this experience affects the strength of USV-evoked On and Off responses across auditory cortical fields by comparing maternal animals to nonmaternal animals (see Materials and Methods). We calculated evoked spike rates of On and Off response on a per-call basis by subtracting an SU's spontaneous rate. We found that, on average, in the core, experience did not affect either On or Off response spike rates (Fig. 6*A*, top; W = 17,091, *Z* = 1.23, *p* = 0.22), although there was considerable variability. Interestingly, though, in A2, maternal animals showed a significantly decreased On response firing rate (Bonferroni-corrected Wilcoxon Rank Sum, W = 2159, *Z* = −4.44, *p* = 9.01 × 10^−6^), but we saw no changes in evoked Off spike rates (Fig. 6*A*, bottom). The same results held if analyses were conducted only for the longest calls (Fig. 1*A*, top row; Bonferroni-corrected Wilcoxon Rank Sum: core, W = 4487, *Z* = 1.32, *p* = 0.18; A2, W = 849, *Z* = −4.89, *p* = 9.6 × 10^−7^). When analyses were performed on a per-SU basis (see Materials and Methods) rather than a per-call basis, A2 still showed significantly decreased On response spike rates in maternal animals (Wilcoxon Rank Sum, *p* < 0.01).

### Prevalence of Off responses to pup USVs increases with experience

Aside from the strength of the evoked response itself, changes may also arise in the proportion of On or Off responses being elicited by USVs. To address this, we computed, on a per-call basis, the proportion of all Exc responses from maternal or nonmaternal SUs that showed an On-Only, On+Off, or Off-Only response to the call. In the core, we found no change in these proportions after maternal experience (Fig. 6*B*, top). However, in A2, the prevalence of Off-Only responses increased significantly (Bonferroni-corrected Fisher's Exact, *p* = 2.37 × 10^−10^), whereas On-Only and On+Off responses decreased significantly (Fig. 6*B*, bottom; Fisher's Exact: On-Only, *p* = 0.00079; On+Off, *p* = 5.7 × 10^−6^). When considering only the longest calls, we found that although On-Only responses became similar between maternal and nonmaternal groups, On+Off and Off-Only responses remained significantly different in A2 (Fisher's Exact: On+Off, *p* = 0.00011; Off-Only, *p* = 0.00013). Even on a per-SU basis using the SU classification from Figure 1*D–F*, Off-Only A2 SUs were still significantly more prevalent in maternal animals (Bonferroni-corrected Fisher's Exact, *p* < 0.005). Finally, if we examine all Off responses by combining On+Off with Off-Only responses, maternal and Nm groups remained significantly different (Fisher's Exact, *p* = 7.90 × 10^−4^). Thus, the proportion of Off responses depends on whether the SU comes from a maternal or Nm animal.

Hence, maternal experience altered both On and Off responses at a population level in A2 more so than in core auditory cortex. Although we found Off responses in both core and A2, A2 was more plastic in terms of how often calls evoked Off responses (Fig. 6*B*), albeit not in how strongly they evoked Off response spiking if they did respond (Fig. 6*A*). The observed plasticity thus tilted the balance in A2 away from responding at the beginning of these short sounds toward signaling as a population more at the calls' terminations, which could better enable a sensitivity to the FMs in pup USVs.

### Specific sFM parameters distinguish stereotypical pup-like and adult-like USVs

In order to explore whether neural plasticity in the sensitivity to FMs in USVs might be adaptive for maternal animals, we next determined whether sFM parameters are distributed so as to acoustically distinguish pup USVs from adult USVs. We applied a nominal logistic regression model to our calls to find parameter ranges for the most stereotyped calls in each category (see Materials and Methods). For each of the six sFM parameters, we determined their optimal weight for calculating all calls' scores to maximize accuracy in predicting ground-truth “pup” and “adult” USV labels (Fig. 2*B*, right). Because of multidimensional overlap of pup and adult USVs in acoustic space, as typically seen for natural vocal categories (Liu et al., 2003), our call classification model was not perfect. Nevertheless, it performed significantly above chance according to a receiver operating characteristics (ROC) analysis (Fig. 2*E*; *p* < 0.0001, area under curve [AUC] = 0.758, 95% CI: 0.754–0.762, bootstrap *N* = 1000). The best performing score threshold was 0.869, where a score >0.869 indicated the call would be classified as having a “pup-like” set of parameters, and all others as “adult-like.” For this choice, the model's sensitivity (true positive rate) was 62.0%, and 1 – specificity (false positive rate) was 16.8%.

Using this model, the overall distributions of our sFM parameters for “pup-like” and “adult-like” calls (Fig. 7*A*) revealed that pup-like calls had systematically lower f_0_, higher A_fm_, longer durations, and higher φ than adult-like calls, suggesting that these parameters were particularly helpful for differentiating stereotyped pup from adult calls. Among the 18 ground-truth pup USVs in our curated set of played back calls, 7 were classified as pup-like by the ideal observer (calls 3, 5, 6, 9, 12, 15, and 18 in Fig. 1*A*), indicating that these were good exemplars of what are the most stereotypical pup calls. Notably, all 6 short-duration, ground-truth pup calls (calls 1, 4, 7, 10, 13, 16) were classified as adult-like, presumably because their short duration was more stereotypical of adult calls. We originally chose our curated set to uniformly span acoustic space (in duration and onset frequency), so it is not surprising that some ground-truth pup calls would have sFM parameters falling into the adult-like space, or vice versa. Nevertheless, our acoustic analysis allowed us to identify those of our 36 exemplars that were most stereotypically pup-like or adult-like in their FM, so that we could examine how auditory cortical SUs respond to these features.

### A2 On and Off response plasticity depends on stereotypical features of pup-like and adult-like USVs

With the finer acoustic classification, we reexamined the decreased On response spiking and increased Off response prevalence for USVs in A2 of maternal animals (Fig. 6). We found that, while suppressed, On response spiking in maternal animals occurred universally for both pup-like and adult-like USVs (Fig. 7*B*, left; Bonferroni-corrected Wilcoxon Rank Sum: pup-like, W = 74,281, *Z* = −10.7, *p* = 1.26 × 10^−26^; adult-like, W = 54,402, *Z* = −9.21, *p* = 3.2 × 10^−20^), the prevalence of On responses decreased specifically for adult-like calls, while the prevalence of Off responses increased specifically for pup-like calls (Fig. 7*B*, right; Bonferroni-corrected Fisher's Exact: On pup-like, *p* = 0.224; On adult-like, *p* = 7.1 × 10^−6^; Off pup-like, *p* = 0.0021; Off adult-like, *p* = 0.144). Hence, in the maternal A2, SUs become more likely to show Off responses precisely for calls that have FMs that are more stereotypically pup-like, highlighting a new form of plasticity in Off responses that depends on the statistical properties of a newly meaningful sound category.

### Response tuning for A_fm_ shifts in the maternal A2 to enhance stereotypical pup USVs

Finally, we asked whether A2 SUs' responses change in their ability to encode FM parameters after maternal experience via changes in their parameter response tuning curves. In a subset of SUs, we selected the curated natural USV that elicited the best response, and then systematically varied A_fm_ around the fixed values of the other 5 sFM parameters that modeled this best call (Fig. 8*A*). The resulting tuning curve for A_fm_ (Fig. 8*B*) was fit with a Gaussian to approximate a best A_fm_ and the point of greatest slope (Max slope).

We found that responses were not always strongest for the specific natural call we played, but that SUs were nevertheless tuned in A_fm_. SUs in the maternal (M) A2 group generally preferred significantly higher A_fm_ values than those in Nm animals or in the core (Fig. 8*C*, top; on a per-call basis, Tukey-Kramer HSD: Nm core vs maternal A2, *p* = 2.44 × 10^−4^; Nm A2 vs maternal A2, *p* = 0.0037; maternal; core vs maternal A2, *p* = 4.18 × 10^−4^). Moreover, maternal A2 SUs also had a significantly larger Max Slope A_fm_ compared with the maternal core or Nm A2 groups (Fig. 8*C*, bottom; *p* < 0.05, Tukey-Kramer HSD). Despite stimuli for different SUs having different starting sFM parameter values since they were centered around different best calls, we did not find systematic differences in those other sFM parameter values between the SUs in each group. Furthermore, since in several cases an SU's best call was one of those with short durations (Fig. 1*A*, bottom row), we used the full response rather than just the Off response, because we could not completely differentiate Off responses from delayed On responses in these cases. However, within the entire cohort of *n* = 24 SUs, very few responded during stimulus playback itself (*n* = 2 maternal core SUs, *n* = 2 Nm core SUs, and *n* = 0 A2 SUs), and our results still held if we only considered responses after the stimulus ended. Our results also held on a per-SU and per-animal basis (using the average best A_fm_ value in each case). Importantly, if we instead compared the best A_fm_ for stimuli varied around a SU's BF instead of around a USV, then we saw no differences across animal groups or region (ANOVA, *p* > 0.05; Tukey Kramer's HSD, *p* > 0.72 for all pairwise comparisons). Hence, our data demonstrate an upward shift in A_fm_ tuning of responses that is specific to tuning around USVs in maternal A2 SUs.

As a last step, we sought to gain some insight into why such tuning changes in A2 might be functionally useful for maternal animals in their encoding of A_fm_ in pup-like sounds. The A_fm_ value of a given USV was correlated with its pup likelihood, as measured by the logistic regression score, so that higher A_fm_ predicted higher pup likelihood scores (Spearman ρ = 0.6766, *p* < 0.0001). The distribution of A_fm_ across pup-like calls showed a larger proportion of high A_fm_ values compared with adult-like calls (Fig. 8*D*). Using ROC analysis, we found that an A_fm_ value of 5009.7 Hz best discriminates pup from adult USVs (AUC = 0.61574, *p* < 0.0001; true positive = 38.8%; false positive = 13.15%). Interestingly, the mean Max Slope A_fm_ value at which the neural response is changing most rapidly and would putatively be the point of best discrimination for the maternal A2 group was 5329 Hz, close to the ideal pup-like versus adult-like USV discrimination point (Fig. 8*C*, bottom), whereas that for Nm A2 was only 1819 Hz. Hence, our results suggest that, with experience, responses in A2 shift their tuning in a key frequency trajectory parameter (A_fm_) in a way that could improve discrimination of a newly meaningful sound category from other natural sounds.

## Discussion

We showed that the strength of Off firing by auditory cortical neurons reflects a sensitivity to FM parameters that delineate the trajectory that a sound took, and this sensitivity can be plastic depending on the behavioral significance of the stimulus. FMs as low as 1/24th octave could significantly alter responses. Although Off responses exist in both primary and secondary auditory cortical fields, those in mouse A2 are particularly plastic after experience with a meaningful sound category whose FMs differentiate one vocal category from another. A specific increase in the prevalence of A2 Off responses to pup-like USVs in maternal mice who have cared for pups is accompanied by a retuning in the A2 sensitivity to ultrasonic FM. Together, these results suggest that a secondary auditory cortical field's Off firing could provide a neural substrate for experience-dependent encoding of behaviorally relevant FMs, such as those in natural, emotional, communicative sounds (Morton, 1977; Ramírez Verdugo, 2006; Rodero, 2011).

The classically defined “offset” response to long-duration sounds is generally implicated for detecting silent gaps in sounds (Weible et al., 2014; Anderson and Linden, 2016). Sound versus onset firing is tuned to different pure tone frequencies (Qin et al., 2007; Fishman and Steinschneider, 2009; Scholl et al., 2010; Anderson and Linden, 2016; Sollini et al., 2018) and spatially localized in distinct ways (He, 2001; Liu et al., 2019). Offsets in the cortex may be mediated through separate synapses than through onsets (Scholl et al., 2010), although postinhibitory rebound (Kopp-Scheinpflug et al., 2018) may generate them in some cases. Most of our “Off” responses likely fall into the category of classic “offset” responses, since they showed a response whose timing would change depending on the duration of the USV. However, because of the short nature of these natural sounds, an important limitation of our study is that we cannot definitively rule out the possibility that delayed onset or sustained firing that coincided with the end of the sounds might have contributed to our observed response. In such cases, those Off responses may be more accurately termed “Late” responses, since spiking begins with a delayed onset while the sound is on and continues well after the sound ends during the Off period. Nevertheless, our results using sounds whose durations are inspired by species-specific vocalizations newly demonstrate how this underappreciated part of the excitatory response to calls is tuned and plastic.

Most earlier studies characterizing “offset” firing used noise or constant tones. Here we found that if a neuron is excited by a particular frequency trajectory, then that response cannot be explained just by a sound with the same static spectrum or by a linear ramp from the initial to final frequency (Figs. 2*D*, 5*C*). While our results confirm that Off responses are sensitive to subtle frequency trajectory modulations, we did not examine how detailed receptive field properties, such as FM velocity or phase sensitivity, might explain responses to specific frequency trajectories. Doing so is complicated since sFM parameters beyond those that we varied work together to define how frequency changes over time. For example, for a fixed duration, increasing f_fm_ can shift when the frequency is increasing and change the phase of the frequency trajectory and even the frequency itself at the end of the sound. Neurons' Off firing can be affected by such f_fm_ changes, even when A_fm_ is not changing (Fig. 4), but deciphering why the firing rate changes with such variations is outside of our scope. Presumably, the temporal integration of excitatory and inhibitory synaptic inputs elicited by a sound's frequency trajectory over the neuron's integration window is critical for generating the FM sensitivity we observed in these short sounds. Computational modeling (Anderson and Linden, 2016; Sollini et al., 2018) will likely be essential for clarifying how this sensitivity arises in the firing after the sound ends, especially given the generally broad pure tone excitatory tuning of many mouse auditory cortical neurons (Linden et al., 2003; Joachimsthaler et al., 2014).

The general encoding of FMs by auditory neurons has been studied previously in a number of ways. Sweep direction selectivity and sweep velocity preference were observed in primary auditory cortex using stimuli with linear or logarithmic unidirectional FM (e.g., Mendelson and Cynader, 1985; Nelken and Versnel, 2000; Tian and Rauschecker, 2004; Razak and Fuzessery, 2006). Phase-locked firing during sFMs in tones lasting on the scale of seconds has also been reported (Whitfield and Evans, 1965; Gaese and Ostwald, 1995; Malone et al., 2014). However, natural sounds often feature much more complex modulation than just unidirectional or sinusoidal sweeps alone (May et al., 1989; Scattoni et al., 2008), yet few studies have systematically explored such combined modulations. Our study was motivated to do so based on the acoustic analysis of mouse USV trajectories (Fig. 7*A*), and the fact that, while unidirectional frequency sweep responsiveness correlates with how well neurons respond to complex trajectories in vocalizations (Carruthers et al., 2013), such responsiveness measures do not capture whether neural activity is actually tuned locally to complex modulations (Figs. 3, 4, 8). By approximating natural calls with our 6-parameter sFM frequency trajectory model (Fig. 2) to explore tuning, we revealed not only sFM parameter tuning around a neuron's BF (Fig. 4), but also graded neural firing as acoustic parameters were varied around natural USVs (Fig. 8*B*). Tuning in FM parameter space might therefore serve as an additional mechanism beyond so-called combination sensitivity (Fitzpatrick et al., 1993; Kanwal et al., 1999; Portfors and Wenstrup, 1999) to create sparse selectivity in some neurons for specific sound frequency trajectories (e.g., Fig. 1*F*).

While most work on auditory cortical neural coding has focused on core fields, much less is understood about the role of noncore fields, and no studies to our knowledge have explored the encoding of complex FM in A2. We observed tuning to sFM acoustic parameters in both core and A2 neurons, with similar general characteristics, allowing us to combine those neural populations in presenting tuning around BF (Fig. 5). This agrees with a recent report (Liu et al., 2019) using wide-scale calcium imaging to observe a strong core as well as A2 Offset response to much longer-duration tones, particularly in the ultrasound range. In our study, in response to natural USVs, evoked On and Off firing rates and prevalence among well-isolated, Exc SUs were comparable between core and A2 (Fig. 6). Moreover, the ∼44% of Exc neurons with Off responses (Fig. 1*B*) we observed is similar to the proportion found in mouse medial geniculate nucleus (Anderson and Linden, 2016), especially in both the ventral and dorsal divisions, which project, respectively, to core and A2 in the mouse (Llano and Sherman, 2008). Hence, extending what has been known for pure tone coding in subcortical auditory areas (Suga, 1964; He et al., 1997), our results confirm that Offset responses are prevalent in both primary and higher-order auditory cortical fields. Furthermore, our study newly suggests that such responses throughout the auditory system may be particularly sensitive to FM and behavioral experience.

Indeed, by combining the mouse maternal model for naturally increasing the behavioral relevance of vocal categories (Dunlap and Liu, 2018) with a parameterization of USV frequency trajectories (Fig. 2*A*), we discovered a potential functional role that A2 offset responses play in encoding FMs in meaningful sound categories. Gaining experience caring for mouse pups correlated with weakened A2 On responses elicited by USVs and significantly increased A2 prevalence of USV-evoked Off-Only responses (Fig. 6), an effect that specifically arose for those USVs with more pup-like frequency trajectories (Fig. 7*B*). By testing A_fm_ tuning around natural USVs, we concluded that only in A2, and only for USVs, was there a significant change from Nm to maternal mice in the tuning to USV frequency trajectory parameters (Fig. 8*C*). Although large-scale changes in tuning were not seen here in the core, plasticity within projection-specific or physiologically distinct subsets of core neurons, as has been previously reported (Shepard et al., 2015), may occur on a smaller scale.

Finally, the fact that representational plasticity in FM encoding in SUs emerges at the level of A2 could reflect its role as a critical interface between veridical sensory encoding in earlier auditory stages and more perceptually relevant encoding to drive behavioral responses to the naturally variable exemplars of a meaningful sound category (Schneider and Woolley, 2013; Kuchibhotla and Bathellier, 2018). There is some evidence that nonprimary rodent auditory cortex can be more invariant compared with primary auditory cortex to acoustic distortions of natural calls (Carruthers et al., 2015). Primate secondary auditory cortex can also exhibit more categorical responses to trained complex sound categories (Tsunada et al., 2011) and is thought to lie along a hierarchical pathway that produces progressively more categorical responses (Leaver and Rauschecker, 2010). At a more detailed circuit level though, since A2 receives input both from core auditory cortex (Covic and Sherman, 2011) as well as the dorsal division of the medial geniculate (Llano and Sherman, 2008), which exhibits traditional offset responses (He, 2001; Anderson and Linden, 2016), it remains to be seen whether the site of plasticity underlying the emergence of more A2 Off responses after maternal experience lies within auditory cortex or at the thalamic level. Nevertheless, by responding to more pup-like frequency trajectories after pup care experience, the maternal A2 may help create a neural representation that is more tolerant of variability in natural call trajectories, so that presumed downstream limbic areas, such as the amygdala (LeDoux et al., 1991; Cambiaghi et al., 2016), can more categorically drive subcortical circuits for maternal responsiveness (Banerjee and Liu, 2013). Perhaps in a similar way, intonation differences between emotionally distinct sounds (Banziger and Scherer, 2005; Zatorre and Baum, 2012) may also be processed through A2 to generate intrinsic or learned physiological responses.
